# Factors Associated with Quit Interest and Quit Attempts among Young Adult JUUL Users

**DOI:** 10.3390/ijerph19031403

**Published:** 2022-01-27

**Authors:** Mahmood A. Alalwan, Jill M. Singer, Megan E. Roberts

**Affiliations:** 1 Division of Epidemiology, College of Public Health, The Ohio State University, Columbus, OH 43210, USA; 2 Division of Health Behavior and Health Promotion, College of Public Health, The Ohio State University, Columbus, OH 43210, USA; singer.159@osu.edu (J.M.S.); Roberts.1558@osu.edu (M.E.R.)

**Keywords:** e-cigarette, JUUL, young adult, quit attempt, quit interest, cessation

## Abstract

Despite reports suggesting young people are interested in quitting e-cigarettes, little work has examined predictors of quit outcomes. This study aimed to identify factors associated with quit outcomes among JUUL e-cigarette users in a longitudinal sample of young adults. We assessed undergraduate past-30-day JUUL users during autumn 2018 (*N* = 225); Our outcomes included short-term quit attempts and interest (spring 2019), and long-term quit attempts (spring 2020). We used logistic regression to examine the associations between our outcomes and JUUL use characteristics, other tobacco use, and sociodemographic factors. Findings indicated 76% of users were interested in quitting JUUL, and more than 40% reported a quit attempt. Quit outcomes were not related to sociodemographics. Short-term quit outcomes were more likely among freshmen and less likely among recent cigarillo users. Heavy JUUL users were more likely to report short- and long-term quit attempts, but JUUL device owners were less likely to report short- and long-term quit attempts. Higher nicotine dependence reduced the likelihood of a long-term quit attempt. There is a need for policy level actions that address tobacco control among this population. Findings suggest a range of unique factors that can inform such policies and programs to curb young adult e-cigarette use.

## 1. Introduction

In the United States, e-cigarette use has increased dramatically over the last few years, particularly among young people. Nationally, past 30-day (current) e-cigarette use surged from 1.5% in 2011 to 19.6% in 2020 among high school students and was their most widely used tobacco product [1,2]. Similarly, young adults showed widespread use of e-cigarettes. A recent national survey estimated the prevalence of current e-cigarette use among young adults aged 18–24 years at 7.6%, the highest prevalence compared to all other adult age groups, including middle-aged and older adults [3].

The high prevalence of e-cigarette use was likely precipitated by JUUL, an e-cigarette brand that revolutionized the market with a high-tech design resembling a USB flash drive. In a relatively short period, JUUL comprised over 70% of the e-cigarette market share [4]. The popularity of this particular brand seems to be particularly high among young people, with an estimated prevalence of 13% among 18–20 years old and 8.2% among 21–24 years old [4,5,6,7,8]. This may be partly due to the nicotine salt used in JUUL’s e-liquids, which allows for higher nicotine delivery with less irritation and better sensory experience compared to older e-cigarettes and e-liquids that used free-base nicotine [9], prompting speculations of its high abuse liability.

Despite the high prevalence, popularity, and potential abuse liability of JUUL, research surrounding JUUL cessation (or any e-cigarette cessation) is still developing. A few reports and anecdotal evidence indicate a desire among JUUL users to quit [10,11,12,13,14,15,16]. For example, cross-sectional studies showed nearly half or more of young adult e-cigarette users reported a quit attempt [13,15]. A few studies have also identified barriers or facilitators to e-cigarette cessation, including nicotine dependence, stress, expectancies for e-cigarette use, perceived health risks, social pressure, and environmental factors such as expense, availability, and social acceptability [13,14,15,16]. Additionally, qualitative evidence suggests that e-cigarette cessation interventions should consider motivation strategies, social pressure, and withdrawal symptoms [12]. Nevertheless, very little work has examined quit outcomes related to JUUL, including quit interest and quit attempts [13]. The aim of the present study was to identify factors associated with quit interest and quit attempts among JUUL users in a longitudinal sample of young adults.

## 2. Materials and Methods

### 2.1. Participants and Recruitment

Our data were from a cohort study examining tobacco use among undergraduates at a large Midwestern University. Before the start of the 2016 autumn semester, our research team contacted 1000 incoming freshmen, who were a random sample of the incoming first-year students aged 18 or older, stratified by first-generation college status, gender, and in- versus out-of-state family residence. An email with a link to an online survey was sent to these students, inviting them to participate in a research study on health behaviors, which yielded 529 respondents. We re-contacted these students in their third year, before the autumn semester in 2018. Simultaneously, we contacted a new random sample of 1000 freshmen, which were recruited in the same way as described above, which yielded 611 new freshmen respondents. The University’s Institutional Review Board approved all study methods. Surveys were sent to both cohorts in August, September, and December 2018 (autumn), as well as March and June (spring) 2019. A final follow-up survey was sent in March 2020. Our study population of current JUUL users included those who reported past-30-day JUUL use at any timepoint in autumn 2018 and who completed at least one survey during spring 2019.

### 2.2. Procedures

At enrollment (2016 or 2018), participants completed an online consent form, followed by a 15-min survey. The initial survey contained questions related to the participants’ ever and past 30-day use of various tobacco products, including e-cigarettes. To address the national surge in JUUL use that occurred in 2018, we added new questions to assess JUUL use specifically during the 2018–2019 academic year.

### 2.3. Variables

#### 2.3.1. Outcomes

The main outcomes of interest in this analysis were short- and long term quit attempts, and short-term quit interest.

Short- and long-term quit attempt status were defined as any reported attempt to quit JUUL during spring 2019 and spring 2020, respectively. At both time points, participants were asked “What best describes your current intentions regarding quitting JUUL?” Participants who selected response options of “currently undergoing a quit attempt”, or “I have already quit”, and who also reported no JUUL use in the past 30 days, were coded as making a quit attempt.

Short-term quit interest status was defined as any reported interest in quitting JUUL during spring 2019. Participants were asked “Have you ever seriously considered quitting JUUL?” Response options were “yes” (coded as having an interest in quitting) and “no” (coded as no interest in quitting). Participants undergoing a quit attempt were excluded from coding for this variable.

#### 2.3.2. Factors Associated with Short-Term Outcomes

Factors expected to be associated with short-term quit attempt and quit interest were all measured in autumn 2018. Factors related to characteristics of JUUL use included owning a JUUL device, heaviness of use, JUUL use frequency, and having friends who use JUUL. Heaviness of use was assessed with the question “Think about your JUUL use over the past 30 days. If you were using a JUUL by yourself without sharing, how long would it take you to finish one pod?” Responses were dichotomized as “less than 1 month” versus “1 month or more”. JUUL use frequency was assessed with the question “In the past 30 days, on how many days did you use JUUL?” Responses were dichotomized as “10 days or less” versus “more than 10 days”. Friends who use JUUL was coded into three levels: “none-a few”, “some”, or “most-all”. When questions were asked on more than one autumn survey, the most recent participant response was used.

Factors related to other tobacco use included any past-30-day use reported during autumn for cigarettes (yes/no), cigars (yes/no), cigarillos (yes/no), hookah (yes/no), and smokeless tobacco (yes/no).

Risk perceptions of JUUL use were assessed using two questions. For the first, we asked “Compared to one pack of cigarettes, how much nicotine do you think is in one JUUL pod?” with the following response options: “there is more nicotine in the cigarette pack”, “there is more nicotine in the JUUL pod”, or “they are about the same”. For the second, we asked “Compared to one pack of cigarettes, how dangerous is it to vape one JUUL pod?” with the following response options: “cigarettes are more dangerous”, “JUULs are more dangerous”, or “they are about the same”. The most recent responses in autumn were used.

#### 2.3.3. Factors Associated with Long-Term Outcomes

To predict long-term quit attempt, we used factors related to characteristics of JUUL use, other tobacco use, and risk perceptions of JUUL use measured in autumn 2019, using the same definitions as described above. We also included JUUL flavor, which was defined as “mint”, “fruit”, and “other flavors”. Additionally, we included scores from the Hooked on Nicotine Checklist (HONC), the Wisconsin Smoking Withdrawal Scale (WSWS), and the E-cigarette Dependence Scale (EDS), measured in autumn 2019 as continuous variables.

#### 2.3.4. Sociodemographic Factors

Social and demographic factors were used to predict short-term quit attempt, short-term quit interest, and long-term quit attempt. These factors included gender, race/ethnicity, and socioeconomic status (SES), which were measured when participants completed their baseline survey. Students’ cohort was defined as “freshmen” or “juniors” based on their year in autumn 2018. We measured SES in terms of three indicators: social class growing up, current social class [17], and parent education (separately assessed for each parent). Our measure of SES was created by calculating and aggregating a z-score for each of these four indicators.

### 2.4. Statistical Analysis

To describe our sample, we presented the characteristics of our study sample using counts and percentages. We also used Chi square tests for categorical variables and two-sample *t*-tests/Wilcoxon Rank Sum tests for continuous variables to describe the associations of each factor of interest with the quit outcomes.

We used separate logistic regression models for each outcome of interest. In general, we used 10 outcome events per predictor variable in our models. To build our models, we used purposeful selection to identify significant factors [18,19]. Specifically, we started with univariable logistic regression analyses for each predictor, with significance defined at *p* < 0.20, followed by a multivariable model with all significant factors. A variable was kept in the multivariable model at *p* < 0.05 or if its removal induced a coefficient estimate change >20% in other variables. Next, we added back the variables excluded from the univariable analyses to the multivariable model, one at a time, and each was assessed for statistical significance at *p* < 0.05. Due to the low sample size and to correct for potential overfitting, we used a bootstrap estimator to calculate confidence intervals [20]. Finally, we checked model assumptions and assessed for potential interactions. All analyses were conducted using SAS 9.4 (Cary, NC, USA).

## 3. Results

### 3.1. Descriptive Statistics

Our final analytical sample included 225 current JUUL users. Of those, 83% were white, 58% female, and 60% freshmen. Nearly all (213, 95%) respondents reported their short-term quit attempt status (spring 19), 139 (62%) reported their short-term quit interest (spring 19), and 163 (72%) reported their long-term quit attempt status (spring 20). There were no differences in race/ethnicity (*p* = 0.89), gender (*p* = 0.38), or SES scores (*p* = 0.50) between the two cohorts. Table 1 and Table 2 show the descriptive characteristics of our sample by short- and long-term outcomes.

About 43% of JUUL users reported a short-term quit attempt. Race/ethnicity (*p* = 0.61), gender (*p* = 0.14), and cohort (*p* = 0.07) did not differ by short-term quit attempt status. Approximately 8% of those who made a short-term quit attempt had recently used cigarillos, compared to 21% of those who did not make an attempt (*p* < 0.01). Additionally, about 4% had recently used cigarettes and 2% had recently used smokeless tobacco among those who made a short-term quit attempt, compared to 21% (*p* < 0.01) and 11% (*p* = 0.02) of those who did not make an attempt, respectively. Of those who made a short-term quit attempt, 12% owned a JUUL device and 78% took a month or more to finish a JUUL pod, compared to 37% and 32% (*p* < 0.01) among those who did not make an attempt, respectively. Over 31% of those making a short-term quit attempt indicated there was more nicotine in JUUL than cigarettes, compared to 20% among those who did not make an attempt (*p* = 0.09). The mean SES scores were similar for those who made and did not make a short-term quit attempt (*p* = 0.73).

Approximately 76% of JUUL users were interested in quitting JUUL. Race/ethnicity (*p* = 0.12), gender (*p* = 0.33), and cohort (*p* = 0.07) were similar for those with and without quit interest. Approximately 15% of those who had quit interest had recently used cigarillos, compared to 35% of those who were not interested (*p* = 0.01). There was no difference in heaviness of use (*p* = 0.63) and having friends who used JUUL (*p* = 0.40) between those with and without quit interest. Nineteen percent of those with quit interest indicated there was more nicotine in JUUL than cigarettes, compared to 24% among those who were not interested (*p* = 0.85). The mean SES scores were similar for those with and without quit interest (*p* = 0.95).

About 50% of those who reported their long-term quit attempt status made a quit attempt. Race/ethnicity (*p* = 0.26), gender (*p* = 0.86), and cohort (*p* = 0.31) did not differ by long-term quit attempt status. Approximately 5% of those who made a long-term quit attempt had recently used cigarillos, compared to 13% of those who did not make an attempt (*p* = 0.06). Of those who made a long-term quit attempt, 15% owned a JUUL device and 68% took a month or more to finish a JUUL pod, compared to 44% and 28% (*p* < 0.01) among those who did not make an attempt, respectively. Over 48% of participants who made a long-term quit attempt indicated there was more nicotine in JUUL than cigarettes, compared to 36% among those who did not make a quit attempt (*p* = 0.02). Distribution of mint (*p* = 0.15), fruit (*p* = 1.00), and other (*p* = 0.57) flavors were similar among those who made and did not make a long-term quit attempt, with mint being the most common flavor used. The mean scores of HONC (*p* < 0.01), WSWS (*p* < 0.01), and EDS (*p* < 0.01) tended to be lower among those who made a long-term quit attempt, and quitters had higher mean SES scores (*p* = 0.49).

### 3.2. Logistic Regressions

Our multivariable logistic regression analyses showed cohort, heaviness of use, owning a JUUL, and recent cigarillo use were significantly associated with making a short-term quit attempt (Table 3). The odds of a short-term quit attempt for freshmen and those who took a month or more to finish a JUUL pod were 113% (95% CI 1.06–4.90) and 500% (95% CI 3.14–14.0) higher, compared to juniors and those who took less than a month to finish a JUUL pod, respectively. The odds of a short-term quit attempt for JUUL device owners and recent cigarillo users were 69% (95% CI 0.10–0.76) and 75% (95% CI 0.05–0.69) lower, compared to those who did not own JUUL devices and non-recent cigarillo users, respectively.

Cohort and prior recent cigarillo use were significantly associated with short-term quit interest (Table 3). The odds of short-term quit interest for freshmen were 125% (95% CI 1.00–5.63) more likely, compared to juniors. The odds of short-term quit interest for recent cigarillo users were 70% (95% CI 0.12–0.77) less likely, compared to non-recent cigarillo users.

Heaviness of JUUL use, owning a JUUL, and WSWS score were significantly associated with long-term quit attempt (Table 4). The odds of long-term quit attempt for participants who took more than a month to finish a JUUL pod were 227% (95% CI 1.58–7.48) higher than those who took less than a month to finish a JUUL pod. For JUUL device owners, the odds of a long-term quit attempt were 57% (95% CI 0.16–1.07) lower, compared to those who did not own a JUUL device. For every 1 unit increase in WSWS score, the odds of long-term quit attempt decreased by 48% (95% CI 0.28–0.88).

## 4. Discussion

In our longitudinal sample of young adults, over three-quarters (76%) of JUUL users expressed an interest in quitting JUUL. Moreover, 43% reported a quit attempt in our short-term outcomes and 50% reported a quit attempt in our long-term outcomes. These are clear indicators of a desire to quit JUUL among this population.

Our analyses further indicated that short-term quit attempts and quit interest were more likely among freshmen than juniors, and less likely among recent cigarillo users than non-users. JUUL users who took more than a month to finish a JUUL pod were more likely to report short- or long-term quit attempts, compared to those who took less than a month to finish a JUUL pod. Those who owned a JUUL device were less likely to report short- or long-term quit attempts. A higher WSWS score significantly reduced the likelihood of a long-term quit attempt.

To our knowledge, this study is the first to report factors prospectively associated with JUUL quit attempts and quit interest using a longitudinal sample. This study found about half of current JUUL users reported a quit attempt, consistent with previous cross-sectional studies [13,15]. Consistent with our findings, Pulvers and colleagues [13] found no associations between JUUL quit attempts and gender, race/ethnicity, or SES, suggesting that quit attempts were widely distributed among the JUUL user population. However, our study found cohort differences were associated with the short-term quit outcomes, which may reflect a shorter length of use and lower nicotine dependence among freshmen. Unlike our study, Pulvers and colleagues [13] found no associations between cohort or other tobacco use with JUUL quit attempt. This could be explained by differences in variables definitions. Specifically, their definition of cohort, which was freshman/sophomore versus junior/senior/fifth year or higher, combined several categories, possibly grouping diverse participants.

### 4.1. Implications

Consistent with recent literature [11,13], our findings show that quit attempts and quit interest among young adult JUUL users are quite common. These findings indicate the need for e-cigarette cessation support for young adults. Our study adds to the literature, a range of unique factors associated with JUUL quit attempts and quit interest among this population. These factors can inform and guide current cessation programs to support JUUL cessation efforts and prevent relapse among young adults, a population characterized by high JUUL use. In particular, cessation programs may promote JUUL quit attempts and quit interest with proper management of cravings and other nicotine withdrawal symptoms. Our findings also suggest that harm perceptions of JUUL may not play an essential role in quitting JUUL among young adults and may be less emphasized.

Extant literature on cigarette smoking cessation indicates similar factors related to cessation efforts, as found in the present study. For instance, daily smokers [21] and smokers with lower nicotine dependence [22] were more likely to quit smoking, while smokers with higher heaviness of smoking [23] were more likely to relapse during the first month of a quit attempt. The fact that we found a similar pattern of findings for e-cigarettes is important, as it suggests that some elements of cigarette cessation programs may be similarly successful for e-cigarette cessation programs.

Our findings suggest the need for policy level actions that address tobacco control among young adult current JUUL users. Given the desire to quit JUUL use among many undergraduates, colleges and universities should consider offering or expanding cessation treatments at student health centers. In addition, they should review their campus smoke-free policies to ensure e-cigarettes are included, offer educational interventions for first-year students at student orientations, and raise awareness of the campus-based resources for students experimenting with e-cigarettes. Experts also recommend broader-level prevention and intervention efforts. For example, at local and state levels, policymakers, in collaboration with colleges and universities, should reduce the e-cigarette retailer density near campuses [24,25]. Additionally, they should consider a tax increase on e-cigarette devices and their refills, which may curb owning a JUUL or other e-cigarette devices or impact the heaviness of use to promote quit attempts and quit interest among this population. Tax and retailer density policies should be implemented with careful evaluation of potential unintended effects, like an increase in other tobacco products use, such as cigarettes [26]. Thus, it is important to carefully consider measures for mitigating such unintended effects, such as increasing the availability and accessibility of tobacco dependence screening and treatment programs by collaborating with local and state tobacco control advocates.

### 4.2. Strengths and Limitations

One of the most important strengths of this study is its longitudinal design, which helps suggest a temporal association between the examined factors and JUUL quit outcomes. Lack of temporal clarity is a major limitation for studies investigating quit related outcomes and e-cigarette use in observational studies. Our study population was drawn from random samples of two cohorts followed prospectively. Assessment of JUUL dependence was wide-ranging, using the HONC, WSWS, and EDS. Nevertheless, our study had several limitations. Race/ethnicity groups were dichotomized into “white” versus “other race/ethnicity” due to the small number of minority participants. This categorization grouped diverse participants into one category, which may have impacted our ability to make conclusions about JUUL use and cessation-related characteristics across different races/ethnicities. Although our specific measures of quit attempts were adapted from established items in the field of cigarette cessation research, they were not validated for JUUL/e-cigarettes. Moreover, items tested in our models were not theory-informed, but rather based on known risk factors for adolescent/young adult use of other types of tobacco products. Due to the exploratory nature of the study and its small sample size, we did not adjust for multiple comparisons. Larger theory-informed cohort studies are needed to replicate the findings and explore additional constructs (such as quit motivation) among other popular e-cigarette users, especially with the rapidly changing e-cigarette market in the United States. Additionally, this study was limited to one university in one region of the United States, which may limit the generalizability of our findings to other young adults who are not in college, from different regions, or who use other brands of e-cigarettes. Lastly, we did not examine quit outcomes among exclusive e-cigarette users, which may further limit the applicability of our findings to these users.

## 5. Conclusions

JUUL use is prevalent among young adults, but a large proportion of these users have an interest in quitting and are making quit attempts. This emphasizes the high need for tobacco control cessation interventions and policy level actions for this population. The present study suggests some unique factors associated with JUUL quit attempts and quit interest that may inform cessation programs and guide policies targeting this population. Future studies should examine cessation-related characteristics among exclusive e-cigarette users and dual product young adult users.

## Figures and Tables

**Table 1:** Descriptive characteristics of current JUUL users by short-term quit outcomes.

	Short-Term Quit Attempt (*N* = 213)					Short-Term Quit Interest (*N* = 139)				
	No		Yes			No		Yes		
	*n*	%	*n*	%	*p* Value ^†^	*n*	%	*n*	%	*p* Value ^†^
**Race Ethnicity**										
White	105	86.1	76	83.5	0.607	26	76.5	92	87.6	0.115
Other	17	13.9	15	16.5		8	23.5	13	12.4	
**Gender**										
Female	65	53.3	57	63.3	0.143	16	47.1	59	56.7	0.326
Male	57	46.7	33	36.7		18	52.9	45	43.3	
Missing			1					1		
**Cohort**										
Juniors	55	45.1	30	33.0	0.074	18	52.9	37	35.2	0.067
Freshmen	67	54.9	61	67.0		16	47.1	68	64.8	
**Autumn 18 Recent Cigarette Use**										
No	97	79.5	87	95.6	0.001	29	85.3	84	80.0	0.491
Yes	25	20.5	4	4.4		5	14.7	21	20.0	
**Autumn 18 Recent Cigar Use**										
No	101	82.8	79	86.8	0.422	31	91.2	85	81.0	0.163
Yes	21	17.2	12	13.2		3	8.8	20	19.1	
**Autumn 18 Recent Cigarillo Use**										
No	96	78.7	84	92.3	0.007	22	64.7	89	84.8	0.011
Yes	26	21.3	7	7.7		12	35.3	16	15.2	
**Autumn 18 Recent Hookah Use**										
No	109	89.3	85	93.4	0.304	31	91.2	94	89.5	0.781
Yes	13	10.7	6	6.6		3	8.8	11	10.5	
**Autumn 18 Recent Smokeless Tobacco Use**										
No	109	89.3	89	97.8	0.017	32	94.1	94	89.5	0.424
Yes	13	10.7	2	2.2		2	5.9	11	10.5	
**Owning JUUL Device**										
No	77	63.1	80	87.9	<0.001	24	70.6	70	66.7	0.671
Yes	45	36.9	11	12.1		10	29.4	35	33.3	
**Heaviness of Use**										
Pod Finished in <1 month	73	67.6	18	22.5	<0.001	18	60.0	61	64.9	0.627
Pod Finished in ≥1 month	35	32.4	62	77.5		12	40.0	33	35.1	
Missing	14		11			4		11		
**Friends who use JUUL**										
None-A few	16	13.1	27	29.7	0.008	6	17.7	16	15.2	0.396
Some	60	49.2	41	45.1		13	38.2	54	51.4	
Most-All	46	37.7	23	25.3		15	44.1	35	33.3	
**Nicotine in JUUL compared to Cigarette Pack**										
The same	77	65.8	46	51.1	0.087	21	61.8	66	66.0	0.848
More in cigarette pack	17	14.5	16	17.8		5	14.7	15	15.0	
More in JUUL pod	23	19.7	28	31.1		8	23.5	19	19.0	
Missing	5		1					5		
**How Dangerous to Vape 1 JUUL Pod**										
The same	38	32.5	26	28.9	0.674	9	26.5	33	31.4	0.775
Cigarette is more dangerous	73	62.4	57	63.3		23	67.7	62	59.1	
JUUL is more dangerous	6	5.1	7	7.8		2	5.9	5	4.8	
Missing	5		1					5	4.8	
	Mean	SD	Mean	SD		Mean	SD	Mean	SD	
**Socioeconomic Status**	0.11	0.69	0.08	0.71	0.732	0.10	0.64	0.10	0.71	0.947

† *p* value for Chi^2^ test for categorical variables. Two-sample *t*-test/Wilcoxon Rank Sum test was used for continuous variables.

**Table 2:** Descriptive Characteristics of Current JUUL users by Long-Term Quit Attempt.

	Long-Term Quit Attempt (*N* = 163)				
	No		Yes		
	*n*	%	*n*	%	*p* Value ^†^
**Race Ethnicity**					
White	73	88.0	67	81.7	0.263
Other	10	12.0	15	18.3	
**Gender**					
Female	47	56.6	47	57.3	0.856
Male	36	43.4	34	41.5	
Missing			1	1.2	
**Cohort**					
Juniors	41	49.4	34	41.5	0.306
Freshmen	42	50.6	48	58.5	
**Autumn 18 Recent Cigarette Use**					
No	63	75.9	74	90.2	0.014
Yes	20	24.1	8	9.8	
**Autumn 18 Recent Cigar Use**					
No	75	90.4	77	93.9	0.399
Yes	8	9.6	5	6.1	
**Autumn 18 Recent Cigarillo Use**					
No	72	86.7	78	95.1	0.061
Yes	11	13.3	4	4.9	
**Autumn 18 Recent Hookah Use**					
No	77	92.8	75	91.5	0.755
Yes	6	7.2	7	8.5	
**Autumn 18 Recent Smokeless Tobacco Use**					
No	75	90.4	81	98.8	0.017
Yes	8	9.6	1	1.2	
**Owning JUUL Device**					
No	46	56.1	69	85.2	<0.001
Yes	36	43.9	12	14.8	
Missing	1		1		
**Heaviness of Use**					
Pod Finished in <1 month	59	72.0	26	32.1	<0.001
Pod Finished in ≥1 month	23	28.0	55	67.9	
Missing	1		1		
**Friends who use JUUL**					
None-A few	15	18.0	19	23.2	0.267
Some	36	43.4	41	50.0	
Most-All	32	38.6	22	26.8	
**Nicotine in JUUL compared to Cigarette Pack**					
The same	46	60.5	31	40.3	0.024
More in cigarette pack	3	3.9	9	11.7	
More in JUUL pod	27	35.5	37	48.1	
Missing	7		5		
**How Dangerous to Vape 1 JUUL Pod**					
The same	21	27.6	28	36.4	0.298
Cigarette is more dangerous	47	61.8	38	49.4	
JUUL is more dangerous	8	10.5	11	14.3	
Missing	7		5		
**Flavor Mint**					
No	11	13.6	18	22.2	0.151
Yes	70	86.4	63	77.8	
Missing	2		1		
**Flavor Fruit**					
No	36	44.4	36	44.4	1.000
Yes	45	55.6	45	55.6	
Missing	2		1		
**Flavor Other**					
No	65	80.2	62	76.5	0.567
Yes	16	19.8	19	23.5	
Missing	2		1		
	**Mean**	**SD**	**Mean**	**SD**	
**Hooked on Nicotine Checklist**	2.07	3.00	0.59	1.59	<0.001
**Wisconsin Smoking Withdrawal Scale**	1.37	0.68	0.98	0.60	<0.001
**E-cigarette Dependence Scale**	2.65	3.36	0.81	1.83	<0.001
**Socioeconomic Status**	0.07	0.65	0.15	0.72	0.485

† *p* value for Chi^2^ test for categorical variables. Two-sample *t*-test/Wilcoxon Rank Sum test was used for continuous variables.

**Table 3:** Factors associated with short-term quit outcomes among current JUUL users.

	Short-Term Quit Attempt						Short-Term Quit Interest					
	OR	95% CI		aOR	95% CI		OR	95% CI		aOR	95% CI	
		LCL	UCL		LCL	UCL		LCL	UCL		LCL	UCL
**Cohort**												
Juniors	Ref			Ref			Ref			Ref		
Freshmen	1.67	0.95	2.93	2.13	1.06	4.90	2.07	0.95	4.53	2.25	1.00	5.63
**Autumn 18 recent Cigarillo use**												
No	Ref			Ref			Ref			Ref		
Yes	0.31	0.13	0.75	0.25	0.05	0.69	0.33	0.14	0.80	0.30	0.12	0.77
**Heaviness of Use (Autumn 18)**												
Pod Finished in <1 month	Ref			Ref								
Pod Finished in ≥1 month	7.18	3.71	13.92	6.00	3.14	14.0						
**Own JUUL**												
No	Ref			Ref								
Yes	0.24	0.11	0.49	0.31	0.10	0.76						

OR: Odds Ratio, aOR: adjusted Odds Ratio, CI: Confidence Interval, LCL: Lower Confidence Limit; UCL, Upper Confidence Limit.

**Table 4:** Factors associated with long-term quit attempts among current JUUL users.

	OR	95% CI		aOR	95% CI	
		LCL	UCL		LCL	UCL
**Heaviness of Use (Spring 19)**						
Pod Finished in <1 month	Ref			Ref		
Pod Finished in ≥1 month	5.43	2.78	10.61	3.27	1.58	7.48
**Own JUUL Spring 19**						
No	Ref			Ref		
Yes	0.22	0.11	0.47	0.43	0.16	1.07
**WSWS**	0.39	0.24	0.66	0.52	0.28	0.88

OR: Odds Ratio, aOR: adjusted Odds Ratio, CI: Confidence Interval, LCL: Lower Confidence Limit; UCL, Upper Confidence Limit.

## Data Availability

The data presented in this study are available upon reasonable request from the senior author (M.E.R.). Per our IRB protocol, the data are not publicly available due to ethical concerns regarding participant confidentiality.
